# Improving the Hydrophilicity of Flexible Polyurethane Foams with Sodium Acrylate Polymer

**DOI:** 10.3390/ma14092197

**Published:** 2021-04-25

**Authors:** Ana M. Borreguero, Javier Zamora, Ignacio Garrido, Manuel Carmona, Juan F. Rodríguez

**Affiliations:** 1 Department of Chemical Engineering, University of Castilla—La Mancha, Av. Camilo José Cela s/n, 13004 Ciudad Real, Spain; anamaria.borreguero@uclm.es (A.M.B.); javierzamora87@gmail.com (J.Z.); manuel.cfranco@uclm.es (M.C.); 2 Department of Applied Mechanics and Engineering Projects, University of Castilla—La Mancha, Av. Carlos III s/n, 45071 Toledo, Spain; Ignacio.Garrido@uclm.es

**Keywords:** polyurethane foams, superabsorbent, absorption capacity, sodium acrylate polymers

## Abstract

Hydrophilic, flexible polyurethane (FPU) foams made from Hypol prepolymers are capable of retaining large amounts of water and saline solutions. The addition of different catalysts and surfactant agents to Hypol JM 5008 prepolymer was assayed to obtain a foam with good structural stability and elasticity. The combination of three catalysts, stannous octoate and two amine-based ones (Tegoamin 33 and Tegoamin BDE), and the surfactant Niax silicone L-620LV allowed to synthesize a foam with a homogeneous cell size distribution, exhibiting the highest saline absorption capacity (2.4 g/gram of foam) and almost complete shape recovery, with up to a 20% of remaining deformation. Then, superabsorbent sodium acrylate polymer (PNaA) was added to the FPU foam up to 8 pph. The urine absorption capacity of the foam was increased about 24.8% by incorporating 6 pph of PNaA, absorbing 17.46 g of saline solution per foam gram, without any negative impact on the rest of the foam properties. All these properties make the synthesized foams suitable for corporal fluids absorption applications in which elasticity and low-density are required.

## 1. Introduction

Flexible polyurethane foams are three-dimensional crosslinked structures with open cells that can easily suffer reversible deformation. This property makes them proper for applications in the upholstery of furniture, mattresses and filled bed products [1,2]. Moreover, some of them also present good water absorption capacity, such as those obtained from the Hypol prepolymers marketed by Dow that can absorb and retain about 20 times their own weight in water [3]. Both properties, elasticity and water absorption are essential in products, such as diapers or sanitary pads. To date, most of these products were made from nonwoven fabrics, paper pulps or spongy urethane resins [4]. However, these materials do not present good elasticity, resulting in uncomfortable for being used in sanitary pads and also, their water absorption needs to be improved. Therefore, some attempts have been made for the substitution of these materials by FPU foams with high water absorption capacity [4,5].

On the other hand, superabsorbent polymers (SAPs) are crosslinked hydrophilic polymers that can absorb and retain large amounts of water and aqueous solutions up to hundreds of times their own weight, being their effectivity in saline solutions also very high [6,7]. Such absorption capacity allows the application of superabsorbent polymers for a wide variety of products, such as disposable diapers, feminine napkins, soils for agriculture and horticulture, absorbent pads, food packaging, artificial snow, controlled delivery of drugs and water-blocking tapes [8,9,10,11,12]. Superabsorbent polymers may have important applications related to the environment as a way to combat desertification or in wastewater and oil spills treatments [9,13,14,15].

In the last decades, the investigation in superabsorbent polymers to improve their absorption capacity has provided many different novel compounds that can be used as superabsorbent polymers, such as sodium acrylate, potassium acrylate, polyacrylamide and also a wide variety of novel composites that combined with superabsorbent polymers improve their properties [16].

Among all the mentioned superabsorbent polymers, sodium acrylate polymers (PNaA) are widely used in aid urine absorption products for babies and old people. They are commonly applied in cellulose materials, but they can result uncomfortable for the users due to the lack of elasticity. Therefore, the combination of flexible PU foams synthesized from hydrophilic prepolymers (Hypol) with sodium acrylate polymers can be an interesting novel alternative for designing more comfortable products with a higher urine absorption capacity. As far as we know, this kind of material has not been reported before.

There are researches that have incorporated superabsorbents, such as sodium acrylate, into previously synthesized polyurethane foams, by filling the previously synthesized foam cells with the superabsorbents [13,17]. In this investigation, part of the novelty lays in the fact that the incorporation of the sodium acrylate polymers into the FPU foams has been carried out by their direct addition into the foaming recipe. Moreover, instead of conventional PU foams, Hypol prepolymer has been used for the synthesis of PU foams. The FPUs containing the PNaA were manufactured in an open recipient to get a foam block. Initially, the best recipe formulation for the hydrophilic FPU foam parting from Hypol prepolymer was studied, using different surfactants and catalysts, since it is known that they determine the final foam structure and properties, but there is a lack of knowledge of the best combination and proportions for this particular case [18,19,20,21,22]. Then, the influence of the sodium acrylate content on the water retention capacity, on the soaking kinetic, and also on the apparent density and flexibility of FPU foams was determined. This way, the best possible foam recipe with the maximum feasible sodium acrylate content was developed.

## 2. Materials and Methods

### 2.1. FPU Foams Synthesis

The flexible PU foams were manufactured by using Tegoamin 33 (a solution of 33% triethylene diamine in dipropyleneglycol), Tegoamin BDE (ethanamine, 2,2’-oxybis[N,N-dimethyl-) (both from Evonik Degussa International AG, Barcelona, Spain), stannous octoate (from Sigma-Aldrich, Madrid, Spain), or a mixture of them as catalysts; SH350 (from Manuel Riesgo SA), Niax silicone L-620LV (from OSI Specialties, Barcelona, Spain) or Tegostab B8404 (from Evonik Degussa International AG, Barcelona, Spain) as surfactants; distillate water; sodium acrylate polymer FAVOR®PAC-230 from Evonik Industries (Barcelona, Spain) and Hypol JM 5008, an NCO-terminated hydrophilic MDI-based prepolymer, from Dow Chemical Company (Navarra, Spain). The mixture of the components was carried out in polypropylene vessels under controlled agitation with a Heidolph stirrer. All the foams are synthesized with the same amount of water and Hypol and with a mass ratio between them of 1 (mass of water = 100 pph of Hypol). Once the masses of the reagents are added, the whole mixture is stirred for 10 s. Then, the foam is led to cure for 24 h at room temperature. The recipes of the different foams are gathered in Table 1, but that named pure foam, which only contains water and Hypol.

In those experiments in which sodium acrylate polymer was added, initially, the corresponding amount of PNaA (from 2.0 to 8.0 pph of Hypol) was mixed with the Hypol prepolymer in a polypropylene vessel and stirred for 20 s; next, the water and corresponding surfactants and catalysts were added, maintaining the agitation for 10 additional seconds. Then, the foam was led to cure for 24 h at room temperature.

### 2.2. Apparent Density Tests

The foams’ apparent density (ρ) was determined according to the standard UNE-EN ISO 845:2006. Three prismatic samples with the dimensions of 50 mm × 50 mm × 25 mm were taken out from each foam using a hot wire device, measuring their twelve edges for calculating their mean and weighting them for the foam densities calculation with Equation (1).

ρ = *m/v* (1)

where *m* and *v* are the mass and the volume of each foam, respectively.

### 2.3. Foam Cell Structure

The foam cell structure was observed using a QUANTA 250 SEM manufactured by FEI Company (Hillsboro, Oregon, EEUU) and equipped with a tungsten filament and a secondary electron detector (LFD). A low vacuum condition of 50 Pa and 10 kV to 15 kV accelerating voltage were used. The average cell size was determined using the software Motic Image Plus (MoticEurope SLU, Barcelona, Spain).

The PNaA microcapsules hydration photos were obtained with an FEI Peltier cooling stage installed inside the SEM. The temperature sample was fixed to 2 °C, and chamber pressure was varied until getting water-saturated atmosphere using SEM environmental mode (ESEM). SEM accelerating voltage was 20 kV.

### 2.4. Urine Retention Capacity

The study of the retention of urine under pressure conditions was carried out according to the standard UNE EN ISO 11948-1:1996. The samples previously used for the density tests were immersed in a saline solution of 9 g/L of NaCl in water at 20 °C for 30 min. After this time, the samples are rolled up and pressed. Then, they are weighed again, and the urine absorption capacity is calculated by using Equation (2):

Q_r_ = (m_2_ − m_1_)/m_1_ (2)

where Q_r_ is the urine retention capacity expressed in grams of solution per gram of foam, m_2_ is the mass of the foam containing the absorbed solution after being pressed, and m_1_ is the mass of the dry foam.

### 2.5. Kinetic of Urine Absorption

The foam samples were introduced in a saline solution of 9 g/L of NaCl in water at 20 °C and taken them out at different times, weighting the foam for each time (m_i_) and determining the amount of urine absorbed per gram of dry foam (m_1_). The measurements ended when the steady-state was reached, and no more solution was absorbed by the foam (m_f_). With these data, the punctual and final saline solution absorption capacities (Q and Q_f_, respectively) were determined according to Equations (3) and (4):

Q = (m_i_ − m_1_)/m_1_ (3)

Q_f_ = (m_f_ − m_1_)/m_1_ (4)

### 2.6. Foam Elasticity

The remaining deformation (rd) after compression tests was determined following the standard UNE EN ISO 1856:2001. The tested foam specimens were prisms of 5 × 5 × 2.5 cm^3^, and each analysis was repeated for 5 samples. Compression tests were performed until the 50% of foam deformation, maintaining this condition for 22 h. After this time, the foams were left to rest for 30 min and then the final foam height was measured. The remaining deformation is calculated by Equation (5):

rd (%) = (d_0_ − d_f_)/d_0_ · 100 (5)

where d_0_ and d_f_ are the initial and final foam heights, respectively.

### 2.7. Water Absorption by SEM

Inside the FEI QUANTA 250 SEM chamber, the sample was cooled on a Peltier stage at low vacuum in environmental mode (ESEM) and aqueous atmosphere. Relative humidity was controlled with temperature sample and chamber pressure. At high relative humidity values, the sample started to absorb water from the chamber atmosphere and from condensation droplets. Photos were taken at different times to observe changes in the sample during water absorption.

### 2.8. Volume of Foam Samples

The change in foam sample volume with water uptake was obtained with a RexconDS3 3D scanner. The foam sample was scanned without water and after reaching saturation.

## 3. Results and Discussion

### 3.1. Determination of the Foam Formulation

**Figure 1:**
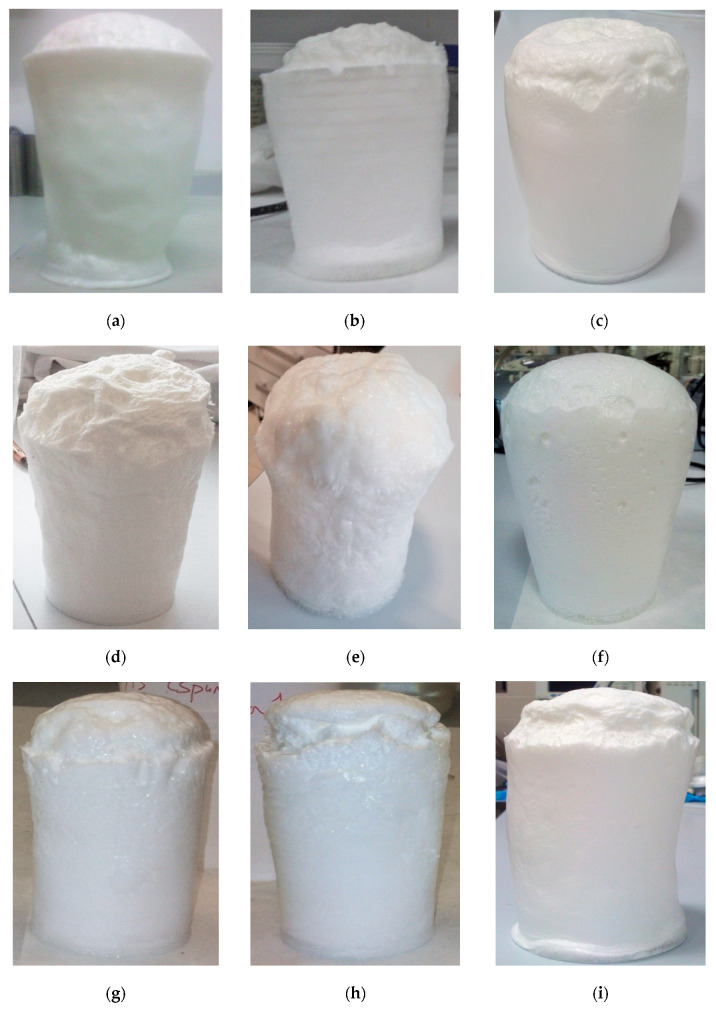
Photos of the synthesized foams: (**a**) 1SH, (**b**) 1.7SH, (**c**) 0.2BDE, (**d**) 0.5BDE, (**e**) 0.2BDE-1SH, (**f**) 0.2BDE-1LV, (**g**) 2.3T33, (**h**) 5T33 and (**i**) 0.05BDE-0.1T33-0.1OC-1.4LV.

The first aim of this research was to find a foam formulation that allowed to obtain a stable foam structure in which incorporates the superabsorbent polymer. The studied foam formulations have been previously shown in Table 1, and photos of the best-synthesized foams are shown in Figure 1.

It was found that the catalyst addition improved the foam structure, reducing cell collapse. Catalysts can promote both of the main foaming reactions, the chain propagation or gelling reaction, between the isocyanate and the polyol, and the blowing reaction, between the isocyanate and water. This way, it is possible to control de cell formation and the cell strength to get a proper structure without cell collapse or shrinkage [23]. Results shown in Figure 1 confirmed the improvement of the foam structure with the catalyst addition, also indicating that for obtaining a stable foam from Hypol, catalyst Tegoamin33 offered the most suitable performance.

Just the surfactant SH250 (experiments 1SH and 1.7SH) avoided the cell collapse in the absence of the catalyst. One of the effects of the surfactant addition in the PU foams synthesis is the decrease of the rate of cell drainage, stabilizing the cell before curing the foam. This is, they avoid or minimize the drain of the liquid into the bubbles and inside the PU lamella, minimizing the cell rupture and foam collapse [24]. However, this effect was only observed in the case of surfactant SH250. The other ones did not generate enough surface tension gradient to avoid the cells rupture.

### 3.2. Apparent Density Test

Apparent densities of foams that did not collapse were calculated according to UNE-EN ISO 845:2006, and their results are collected in Table 2.

According to the apparent density values, the addition of amine-based catalysts (such as Tegoamin BDE or Tegoamin 33) promoted higher foam densities since they favor the crosslinking reaction, being the foam structure formed faster and therefore, lower expansion is achieved. As shown, the catalyst type also affected the foam density, being Tegoamin BDE the one that promoted the highest foam density. These differences in density values were probably due to the relative speed of the blowing and crosslinking reaction.

On the other hand, the surfactant incorporation promoted low foam density values close to those typical for foams based on Hypol prepolymers, which are common in the range of 62 and 72 kg/m^3^ [25]. Moreover, comparing experiments 1SH and 1.7SH, it is observed that the higher the surfactant concentration, the lower the foam density. This behavior agrees with the effect observed by other authors, such as Lim et al. (2008) [19], and it can be justified considering that the surfactants reduce the surface tension of the foaming system, favoring the bubble formation, the blowing agent dispersion and, thus the cell expansion [24].

### 3.3. Saline Solution Retention

**Figure 2:**
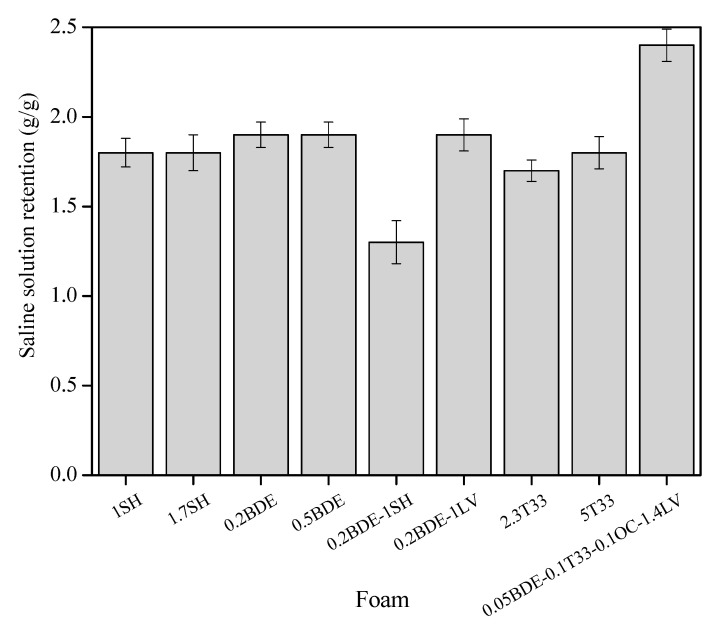
Saline retention capacity of foams based on Hypol prepolymers.

The capacity of saline solution retention was tested according to the standard UNE EN ISO 11948-1:1996, and the data for the studied foams are shown in Figure 2.

The worst behavior was observed for foam 0.2BDE-1SH, which just absorbed 1.3 g of saline solution per gram of dry foam. Most of the foams synthesized had a similar absorption capacity of around 1.8 g of saline solution per gram of foam, but that made using the combination of catalysts and the surfactant agent L-620LV, which exhibited the highest saline solution absorption capacity (2.4 g per gram of dry foam). Hence, this combination of catalysts and using L-620LV as a surfactant agent promoted the formation of a foam presenting good growth, low-density and exhibiting the largest saline solution absorption capacity.

### 3.4. SEM Photography

**Figure 3:**
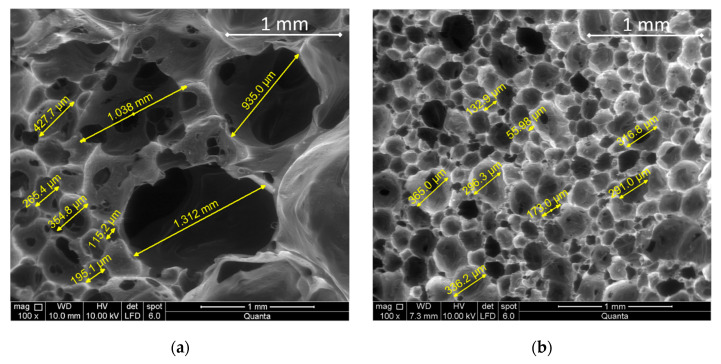
SEM photos at 100× magnification of foams 0.5BDE (**a**) and 0.05BDE-0.1T33-0.1OC-1.4LV (**b**).

To understand the higher saline solution retention of the foam 0.05BDE-0.1T33-0.1OC-1.4LV with respect to the others, its internal structure was compared with that of the foam 0.5BDE, which also showed good stability appearance and density, but worse saline solution absorption. Figure 3 shows SEM photographs of foams 0.5BDE and 0.05BDE-0.1T33-0.1OC-1.4LV.

According to Figure 3a, the foam 0.5BDE presented a very heterogeneous cell size with some big holes of up to 2 mm. On the contrary, Figure 3b, corresponding to the foam 0.05BDE-0.1T33-0.1OC-1.4LV, presented a homogeneous cell size distribution.

**Figure 4:**
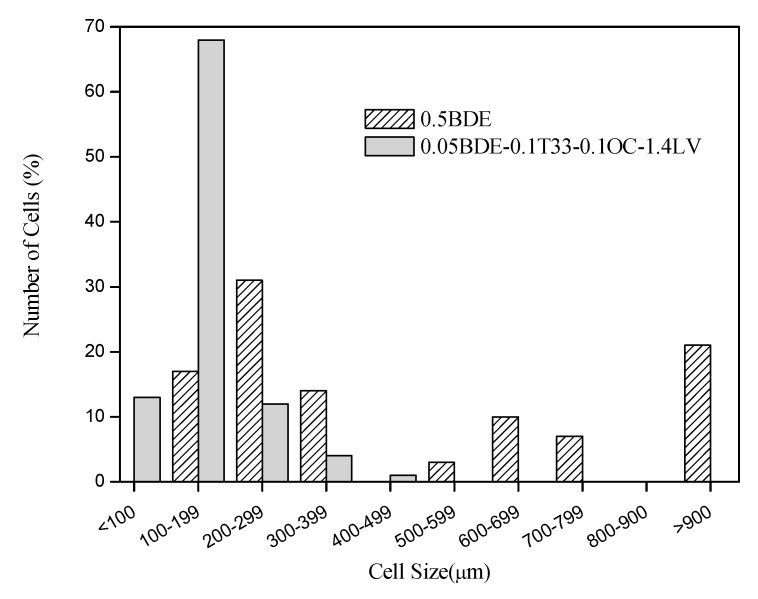
Cell size distribution of foams 0.5BDE and 0.05BDE-0.1T33-0.1OC-1.4LV.

The cell size distributions of both foams were determined by using the software Motic Images Plus to measure the cell sizes of different foam pictures, and they are shown in Figure 4.

As shown in Figure 4, the foam 0.5BDE had more than a 20% of cells higher than 900 μm, while almost the 70% of the cells of foam 0.05BDE-0.1T33-0.1OC-1.4LV had a size within 100–200 μm and the 95% of them were smaller than 300 μm. The higher the cell density (number of cells per area), the higher the surface area for favoring the saline solution uptake; what can be the reason for the better saline solution absorption capacity of the foam 0.05BDE-0.1T33-0.1OC-1.4LV. These results agree with those reported by previous authors that observed higher absorption capacity with the smaller cell size for polyurethane foams [26] and also for other highly absorbent foam types [27]. Moreover, the cell size of foam 0.05BDE-0.1T33-0.1OC-1.4LV is in the range of those from similar products applied in sanitary and wound healing applications [26].

### 3.5. Compression Test

For these tests, foams with different saline solution absorption capacities were selected. In this way, foams 1SH, 0.2BDE, 2.3T33 and 0.05BDE-0.1T33-0.1OC-1.4LV were analyzed.

The compression tests were performed according to the standard UNE EN ISO 1856:2001, observing the remaining deformation of the foams after being compressed at the temperature of 23 °C for 22 h (Table 3).

The best elasticity was observed for foams 1SH and 0.05BDE-0.1T33-0.1OC-1.4LV since they recovered their volume in 100% and 80%, respectively. This property is important for applications, such as disposable diapers or absorbent pads, and, according to these results, it seems to be favored by the presence of the surfactant agent.

### 3.6. Influence of the Sodium Acrylate Polymer (PNaA) Incorporation

**Figure 5:**
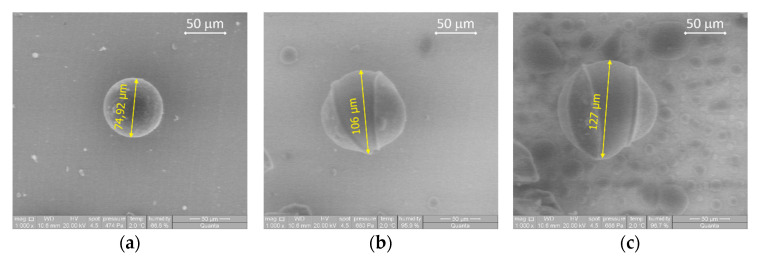

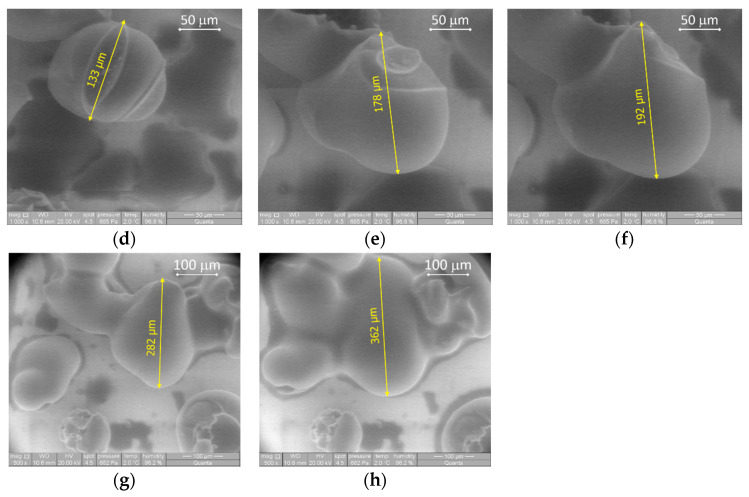
SEM photos of PNaA microcapsules dried (**a**) and with increasing hydration from (**b**–**h**) every 30 s. Photos magnification is 1000× from (**a**–**f**) and 500× in (**g**,**h**).

Taking into account the foams’ properties, the recipe employed for synthesizing the foam 0.05BDE-0.1T33-0.1OC-1.4LV was selected as the best option for developing hydrophilic, flexible PU foams by incorporating sodium acrylate polymer for getting improved saline solution absorption capacities. As commented in the introduction section, the PNaA has a great capacity for water absorption, multiplying their size at least 5 times, as shown in Figure 5. In this figure, it is observed that this material was also able to uptake water from a vapor media saturated with water. The PNaA morphology changed with the content of water from a spherical particle to an amorphous one and gathering with the others around.

Different sodium acrylate polymer quantities from 2 to 8 pph of Hypol were added, studying its content influence on the main foam properties.

### 3.7. Influence of the PNaA Content on the Foam Apparent Density

Table 4 summarizes the data obtained from the apparent density tests performed according to the standard UNE-EN ISO 845:2006.

As shown in Table 4, the foam apparent density was similar to that of the reference foam, even for that with the highest SAP. Thus, the reaction between the isocyanate group and the water for the CO_2_ generation is faster than the absorption of the water by the SAP. On the contrary, the decrease of the blowing agent formation would promote foams with lower final heights and higher densities.

### 3.8. Saline Solution Absorption Test

To study the kinetics of the saline solution absorption by the foams, it was measured for different immersion times as explained in the material and methods section.

**Figure 6:**
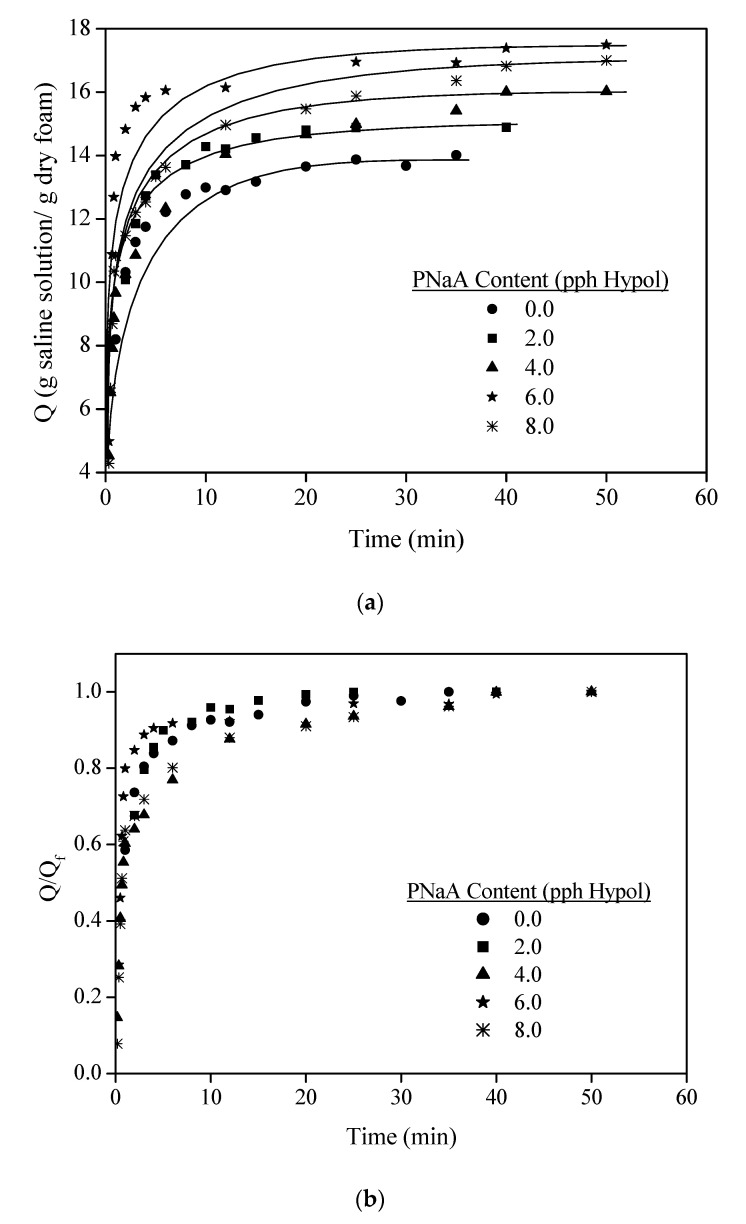
Kinetic curves of saline solution absorption (**a**) and the ratio between the punctual and final saline solution absorption capacities (**b**) of foams with different PNaA contents.

The kinetic curves of saline solution absorption and the ratio between the punctual and final saline solution absorption capacities (Q and Q_f_, respectively) depending on the PNaA content are shown in Figure 6.

According to Figure 6a, all the foams followed similar absorption kinetic and therefore, the phenomena is controlled by the foam structure instead of the superabsorbent concentration. The swelling kinetic presented a first regime characterized by a fast uptake and a second one corresponding to the asymptotic increase of absorption towards the equilibrium, which is typical of highly hydrophilic foams [28]. Moreover, Figure 6b also confirmed that the absorption process was very rapid since all the foams reached 60% of the total saline solution uptake in just one minute and up to 90% in five minutes for the best of them.

The final values of saline solution absorption of foams are shown in Table 5.

**Figure 7:**
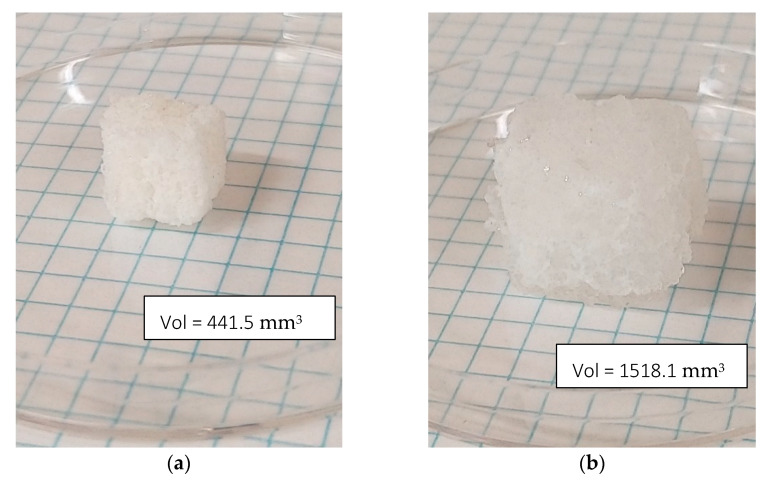

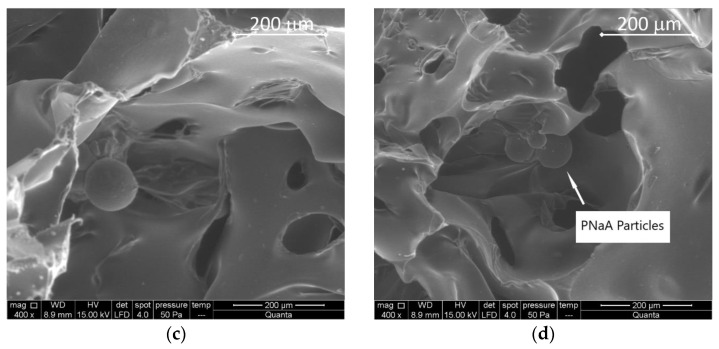
Photos of an 8.0 pph of SAP foam sample before (**a**) and after hydration (**b**). SEM photos at 400\× magnification of an 8.0 pph of SAP foam sample before hydration (**c**) and after hydration (**d**).

As can be seen, the final saline solution absorption capacity improved with the increase of the SAP content up to the addition of 6 g per 100 g of Hypol, showing a saline solution absorption capacity of 24.8% higher than that of the original foam. However, the increase from 6.0 to 8.0 pph of SAP did not improve the absorption capacity, and, therefore, the PNaA content of 6.0 pph seems to be the optimum value for these foams type. It was shown in Figure 5 that the PNaA particle size increased greatly and broke due to the water absorption. This particle increase is higher than the one experimented by the foam due to the water absorption, which is about 3.5 times in volume (Figure 7a,b). Thus, PNaA particles, being embedded and compressed by the foam material, cannot swell freely, limiting the absorption of saline solution and continuing with a spherical shape (Figure 7c,d). Thus, the fact that the optimal value was the 6 pph maybe because, for higher contents, sufficient internal stresses are generated in the foam material to compress the particles, and this does not permit higher swelling of the PNaA and, therefore, it is not able to absorb more saline solution. On the other hand, Santiago et al. found similar optimal concentration values (5.0 wt%) when also using sodium acrylate and sepiolite as superabsorbents, and they suggested that higher superabsorbent contents promoted an increase in the crosslinking density that hampers the saline solution permeation [16]. It also worthy to point out that the maximum saline absorption capacities achieved are higher than those shown by new developed hydrophilic foams, such as those reported by Capezza et al., who combined wheat gluten foams with cellulose nanofibers and genipin, reaching a maximum absorption capacity of 9.9 g_saline solution_/g_foam_ for the case of the wheat gluten foam with just genipin [29].

### 3.9. Influence on the Foam Elasticity

Compression tests were carried out according to the standard UNE EN ISO 1856:2001 to study the influence of the sodium acrylate percentage on the polyurethane foam elasticity, measuring the remaining deformation. Results indicated that, in the same way, that the original foam 0.05BDE-0.1T33-0.1OC-1.4LV, all the foams containing PNaA preserved the elasticity, presenting a remaining deformation of 20%. Therefore, the incorporation of sodium acrylate polymers in the range of the studied contents did not influence the elasticity of the foam, while it allowed increasing their saline solution absorption capacity from 14.02 to 17.49 g of saline solution per gram of foam.

## 4. Conclusions

A catalyst mixture constituted by Tegoamin BDE, Tegoamin 33 and OcSn2 and containing L-620LV as stabilizer allowed to obtain the foam with the highest saline absorption capacity and proper physical and mechanical properties, mainly due to the lower cell size distribution (foam 0.05BDE-0.1T33-0.1OC-1.4LV). The addition of PNaA contents from 2.0 to 8.0 pph of Hypol did not modify the apparent density and final foam elasticity. This indicated that the reaction between the isocyanate and water is faster than the water absorption by the PNaA. Moreover, it was observed that a PNaA content of 6 pph with respect to the Hypol enhanced the foam saline solution absorption capacity by 24.8% and also that a higher content did not improve this property since the PNaA swelling is limited by the foam structure. Thus, the SAP content of 6pph of Hypol was considered as the optimum value for the improvement of the saline solution absorption capacity of this flexible PU foam type. This way, an improved product suitable for being used in urine absorption applications was developed.

## Figures and Tables

**Table 1:** Recipes of the different synthesized foams.

Foam Name	Catalyst			Surfactant		
	Tegoamin BDE	Tegoamin 33	SnOct_2_	SH350	L-620LV	TEGOSTAB
	(pph Hypol)					
1SH	-	-	-	1.0	-	-
1.7SH	-	-	-	1.7	-	-
2LV	-	-	-	-	2.0	-
1TAB	-	-	-	-	-	1.0
0.2BDE	0.2	-	-	-	-	-
0.5BDE	0.5	-	-	-	-	-
0.2BDE-1SH	0.2	-	-	1.0	-	-
0.2BDE-1LV	0.2	-	-	-	1.0	-
2.3T33	-	2.3	-	-	-	-
5T33	-	5.0	-	-	-	-
0.05BDE-0.1T33-0.1OC-1.4LV	0.05	0.1	0.1	-	1.4	-

**Table 2:** Densities of foams with stable structure.

Foam Name	Apparent Density (kg/m^3^)
1SH	66.61 ± 0.83
1.7SH	82.60 ± 0.95
0.2BDE	167.55 ± 1.06
0.5BDE	161.97 ± 1.11
0.2BDE-1SH	81.86 ± 0.48
0.2BDE-1LV	94.40 ± 0.77
2.3T33	128.59 ± 0.94
5T33	129.80 ± 0.61
0.05BDE-0.1T33-0.1OC-1.4LV	97.92 ± 0.67

**Table 3:** Foam remaining deformation was observed after the compression tests.

Foams	Remaining Deformation (%)
1SH	0
0.2BDE	44
2.3T33	46
0.05BDE-0.1T33-0.1OC-1.4LV	20

**Table 4:** Apparent density of the foam 0.05BDE-0.1T33-0.1OC-1.4LV containing different PNaA proportions.

PNaA (pph of Hypol)	Apparent Density (kg/m^3^)
0.0	97.92 ± 0.67
2.0	97.82 ± 1.21
4.0	98.42 ± 0.51
6.0	100.89 ± 0.84
8.0	94.78 ± 1.50

**Table 5:** Final saline solution absorption capacity (Q_f_) of foams incorporating sodium acrylate polymer.

PNaA (pph of Hypol)	Q_f_ (g_saline solution_/g_foam_)
0	14.02
2	14.89
4	16.02
6	17.49
8	17.06

## Data Availability

Data supporting reported results can be provided by the corresponfing author if necessary.
